# Health technology assessments, evidence-based healthcare and health ethics

**DOI:** 10.1590/S1516-31802008000500001

**Published:** 2008-09-04

Health technology assessments imply evaluations of any type of approach or device, drug or medication that is in use or, as would be better, that is yet to be applied to healthcare. At present, there is some confusion between health technology assessments and the application of the original tools for evidence-based medicine, to the point that the terms "evidence-based medicine" and "systematic reviews" were used as the descriptors (key words) in a recent article on the fundamentals of health technology assessments.^1^

Such evaluations in the light of reasonable evidence of efficacy, effectiveness and safety evolve naturally to evaluations of the efficiency of new technology (economic viability) and, furthermore, they also require assessment of the ethics of implementing the subject of the assessment, whether it consists of diagnosis, screening, prevention, prophylaxis or therapy.

The ethical aspects are fundamental.^2^ For example, a kidney donated by an unrelated living individual may function as well as one from a related living donor, but this raises extremely important ethical questions and requires regulation. The principle of non-harmfulness always needs to be taken into account, without forgetting about the patient's right to make decisions regarding the likely risks and benefits of each possible alternative, after receiving appropriate information.

We physicians are becoming increasingly accustomed to obligatory assessments regarding the ethics of research projects, both of clinical and of experimental nature, conducted by research ethics committees within the institutions concerned and at the National Health Board (*Conselho Nacional de Saúde*). With regard to clinical trials, it is mandatory to obtain approval from the local ethics committee and to register the trial. We also take the view that it is of fundamental importance that, when the authorization for the research is granted, the researchers and the entities funding the project should take on an obligation to fulfill the commitment to publish the results from the clinical research. Through this, the promise made to patients who agree to participate in clinical studies after being convinced (by the researchers) that they will be running a calculated risk, with the purpose of contributing towards the improvement of scientific knowledge of use for humanity, can in fact be honored. Failure to publish is a failure to fulfill a tacit agreement with the participants, which could be considered unethical.

However, beyond this, with regard to producing syntheses of the technology assessments accomplished, after incorporating the evidence relating to effectiveness, efficiency and safety, the new knowledge constitutes precious material to be integrated into the evidence-based clinical guidelines and will be tested like new products. The body of good evidence can then be incorporated into the guidelines. Since these guidelines are composed of complex technology, a new technology assessment is thus required and, in our view, this should include establishing a rigorous consensus among the widest range of different professionals, specialists, funding providers, healthcare economists and patient representatives. Finally, there should be rigorous assessment of the ethical implications of applying the guideline. Through this, conflicts of interest and deleterious aspects of any type of corporatism can be reduced and decision-making can be more impartially centered on the **fundamental human interests** that are the motive for all the research accomplished.

In summary, approval needs to be obtained not only for the bioethical aspects of clinical trials but also for those of technology assessments and clinical guidelines. Participation by specialists within the field in question needs to form part of the consensus behind the corresponding guidelines, which will only achieve good professional adherence when they are based on good quality scientific evidence.
